# Supplementary material for: Analysis of PPARγ Signaling Activity in Psoriasis
Source: Int J Mol Sci. 2021 Aug 10;22(16):8603. doi: 10.3390/ijms22168603 (PMC8395241; doi:10.3390/ijms22168603)
Supplement: Supplementary file 1 [file ijms-22-08603-s001.zip › Supplemental materials_Analysis of PPARg signaling activity in psoriasis/Pathway models/Models images and html files/PPARG expressed in cells assotiated with psoriasis/116081.html]

epidermis


# Tissue epidermis

|  |  |
| --- | --- |
| URN | urn:agi-ncimtissue:C0014520 |
| Total Entities | 9 |
| Connectivity | 6965 |
| Name | epidermis |

---

|  |  |
| --- | --- |
| ChildConcepts | interfollicular epidermis |
|  | stratum basale |
|  | dermoepidermal junction |
|  | stratum lucidum (epidermis) |
|  | stratum conjunctum |
|  | stratum disjunctum |
|  | stratum corneum |
|  | stratum granulosum |
|  | stratum spinosum |

---

|  |  |
| --- | --- |
| Pathway | PPARG expressed in cells assotiated with psoriasis |

---

|  |  |
| --- | --- |
| MedScan ID | 6000246 |

---

|  |  |
| --- | --- |
| Alias | epidermal |
|  | epidermis |
|  | epidermides |
|  | epidermial |
|  | epidermises |

---

|  |  |
| --- | --- |
| NCIm ID | C0014520 |

---
